# Microbial Biogeography Along the Gastrointestinal Tract of a Red Panda

**DOI:** 10.3389/fmicb.2018.01411

**Published:** 2018-07-05

**Authors:** Yan Zeng, Dong Zeng, Yi Zhou, Lili Niu, Jiabo Deng, Yang Li, Yang Pu, Yicen Lin, Shuai Xu, Qian Liu, Lvchen Xiong, Mengjia Zhou, Kangcheng Pan, Bo Jing, Xueqin Ni

**Affiliations:** ^1^Animal Microecology Institute, College of Veterinary, Sichuan Agricultural University, Ya’an, China; ^2^Chengdu Wildlife Institute, Chengdu Zoo, Chengdu, China; ^3^Sichuan Animal Science Research Institute, Chengdu, China

**Keywords:** *Ailurus fulgens*, gastrointestinal tract, microbiota, *Escherichia–Shigella*, Illumina HiSeq sequencing

## Abstract

The red panda (*Ailurus fulgens*) is a herbivorous carnivore that is protected worldwide. The gastrointestinal tract (GIT) microbial community has widely acknowledged its vital role in host health, especially in diet digestion; However, no study to date has revealed the GIT microbiota in the red panda. Here, we characterized the microbial biogeographical characteristics in the GIT of a red panda using high-throughput sequencing technology. Significant differences were observed among GIT segments by beta diversity of microbiota, which were divided into four distinct groups: the stomach, small intestine, large intestine, and feces. The stomach and duodenum showed less bacterial diversity, but contained higher bacterial abundance and the most unclassified tags. The number of species in the stomach and small intestine samples was higher than that of the large intestine and fecal samples. A total of 133 core operational taxonomic units were obtained from the GIT samples with 97% sequence identity. Proteobacteria (52.16%), Firmicutes (10.09%), and Bacteroidetes (7.90%) were the predominant phyla in the GIT of the red panda. Interestingly, *Escherichia–Shigella* were largely abundant in the stomach, small intestine, and feces whereas the abundance of *Bacteroides* in the large intestine was high. Overall, our study provides a deeper understanding of the gut biogeography of the red panda microbial population. Future research will be important to investigate the microbial culture, metagenomics and metabolism of red panda GIT, especially in *Escherichia–Shigella*.

## Introduction

The red panda (*Ailurus fulgens*) is a vulnerable wildlife species that belongs to the family *Ailuridae*, which is endemic to *Carnivora* (Yu et al., 2011). The species lives mainly in temperate forests in China, Bhutan, India, Burma, and Nepal. Their population is threatened by the climate, diet, and human activity (Deryabina et al., 2015; Princée and Glatston, 2016). A number of serious illnesses have led to death in this species, as investigated in the 20-year survey of captive dead red pandas (Delaski et al., 2015). Surveys show that pneumonia is the most common cause of death in newborns and juveniles, whereas cardiovascular disease, renal disease, and gastrointestinal disease are the common causes of death in the adult and geriatric red pandas. The probability of the survival of red panda has increased with successful captive breeding (Kumar et al., 2016). Improved captive measures, such as nutrition diets, regular veterinary care, and species breeding strategies, can promote the protection and conservation of red panda.

Intestinal microbial research is currently an important means of wildlife protection and conservation (Amato, 2013; Barelli et al., 2015; Stumpf et al., 2016). Microbiota plays a vital role in animal intestinal digestion, immune response, physiology, and disease treatment (Byndloss et al., 2017; Ramos and Hemann, 2017; Zheng et al., 2017). Together with the giant panda, the red panda is a herbivorous carnivore with simple gut morphologies (Ley et al., 2008). Nevertheless, they both specifically eat bamboo and shared 10 pseudogenes associated with digestion (Fei et al., 2017; Hu et al., 2017). Among the main candidate genes of the pseudothumbs, *DYNC2H1* and *PCNT* are mainly related to the absorption of amino acids in bamboo. Several studies have evaluated the faecal microbiota of red pandas and compared them with other wild animals, especially the giant panda (Kong et al., 2014; Li et al., 2015; Nishida and Ochman, 2017). Firmicutes was the predominant phylum found in the red and giant panda faecal, of which the bacteria abundance is extraordinary high in the giant panda. In particular, Proteobacteria was also found to be the second main flora in the red panda faecal. Firmicutes was found to be closely related to the degradation of bamboo fiber (Zhu et al., 2011). However, there is still relatively little research on the Proteobacteria in these animals gastrointestinal tract (GIT). No study has been conducted about the GIT microbiota of the red panda. All previous studies were conducted using faecal samples of red panda.

Our previous study assessed the bacterial diversity of this red panda using the polymerase chain reaction-denaturing gradient gel electrophoresis (DGGE) (Li et al., 2017). Higher bacterial diversity was found in the stomach and large intestine, whereas less bacterial diversity was obtained in the small intestine. Moreover, abundant DGGE bands in the GIT of red panda were identified with most belonging to Firmicutes, whereas the identified bacteria that belong to Proteobacteria were dominant in all segments. To improve our understanding of the microbial community structure and composition in red panda GIT, the Illumina HiSeq sequencing method is required. We hypothesize that the number and species of bacterial populations in the red panda’s GIT is greater than what we know in it’s faecal matter. We predict that Proteobacteria predominates in the GIT of the red panda. Our findings provide the first insight into the gut biogeography of microbial populations in red panda using high-throughput next-generation sequencing technology.

## Materials and Methods

### Ethics Statement

The sample collection of the dead red panda was approved by the Ethical Committee of Animal Care and Use Commission of Chengdu Institute of Wildlife, Chengdu Zoo. Laboratory experiments were approved by the Animal Microecology Institute of Veterinary Medicine, Sichuan Agricultural University.

### Sample Collection

In July 2016, a male, 5-year-old captive red panda was found dying in Chengdu Zoo. The animal was involved in a fight and its tail was docked before death. GIT contents were derived from GIT segments, which include the stomach, small intestine (duodenum, jejunum, and ileum), and large intestine (colon and rectum). The contents at the beginning and end of each segment were discarded. The content samples close to the middle of each segment were mixed. All GIT contents were collected within one day of the death of the red panda. In the same colonial house, faecal samples from other captive red pandas were thoroughly mixed to form a replacement faecal sample for the dead red panda. All samples were collected in accordance with the Sichuan Agricultural University Committee ethics (Certificate No. SYXKchuan 2014-187) regarding the care and use of experimental animals. The samples were placed in sterile tubes, frozen immediately, sent to the lab in 20 min, and stored at -80°C until further analysis.

### DNA Extraction and Sequencing

Microbial genomic DNA was extracted from GIT contents and faecal samples (0.2 g each) using the QIAamp Stool Mini Kit (Qiagen, Germany). DNA concentration and purity were monitored on a Nano Drop spectrophotometer (Nano Drop Technologies, Wilmington, DE, United States) to ensure it is greater than 20 ng/μl and stored at -80°C prior to further analysis.

The V4 hypervariable region of the 16S rRNA gene from microbial genomic DNA was PCR-amplified using 515F (GTGCCAGCMGCCGCGGTAA) and 806R (GGAC TACHVGGGTWTCTAAT) primers with a 6 bp error-correcting barcodes (Caporaso et al., 2011) (**Supplementary Table S1**). PCR reactions were performed in triplicate with 20 μl of a mixture that contains 8 μl DNA, 1 μl each primer, 10 μl Phusion^®^ High-Fidelity PCR Master Mix and 1 μl of ddH_2_O. The following PCR reaction conditions were used: initial denaturation at 98°C for 1 min, 35 cycles of denaturation at 98°C for 10 s, annealing at 55°C for 30 s, and elongation at 72°C for 30 s and then 72°C for 5 min. PCR products were then mixed with the same volume of 1× loading buffer (contained SYBR green) and ran on a 2% agarose gel. PCR products with bright dominant bands of 400–450 bp were mixed at equal density ratios. The mixture PCR product was purified using a Qiagen gel extraction kit (Qiagen, Germany). Sequencing libraries were constructed according to the instructions using the TruSeq^®^ DNA PCR-Free Sample Preparation Kit (Illumina, United States) and indexed by addition codes. Library quality was assessed using a Qubit@2.0 Fluorometer (Thermo Scientific) and Agilent Bioanalyzer 2100 system. Finally, sequencing was performed on the Illumina HiSeq 2500 platform, which generated 250 bp paired-end reads. The original 16S rRNA sequence data was available in the National Center for Biotechnology Information, BioProject ID PRJNA385220 and Sequencing Read Archive (SRP106218^1^).

### Bioinformatics Analysis

The barcode and primer sequences (Caporaso et al., 2011) of paired-end sequencing readings in all samples were first removed. FLASH was used to assemble sample reads (V1.2.7^2^) (Magoč and Salzberg, 2011). Raw tags were filtered (Quality threshold < = 19, default length 3, and continuous high-quality base length greater than 75% tags) to obtain clean tags using QIIME (Version 1.7.0^3^) (Caporaso et al., 2010). To improve the quality of the analysis, a gold database^4^ and UCHIME algorithm^5^ (Edgar et al., 2011) were used to compare tags and remove chimeric sequences (**Supplementary Table S1**). Uparse software (Version 7.0.1001^6^) was used to cluster valid tags to obtain operational taxonomic units (OTUs) at 97% similarity level (Edgar, 2013). After removing the chloroplast and mitochondrial reads, species annotation (Threshold 0.8-1) was performed by the SSU rRNA database of SILVA^7^ (Wang et al., 2007) and mothur (v 1.32) (Schloss et al., 2009). The classification level included the kingdom, phylum, class, order, family, genus, and species. Multiple sequence alignment analysis was performed using MUSCLE (Version 3.8.31^8^).

Alpha and beta diversity were analyzed using the QIIME (Version 1.7.0) (Caporaso et al., 2010) and visualized using R software (Version 2.15.3) (McMurdie and Holmes, 2013). With rarefaction at each sampling depth, alpha diversity included Shannon index, Simpson index, Observed-species, Good’s coverage, Chao1, and ACE. Chao1 and ACE were obtained and show the community richness of the red panda GIT. The Shannon and Simpson indices revealed community diversity. The principal component analysis (PCA) of unweighted UniFrac distances was constructed (Lozupone et al., 2011). To compare the differences between the three groups, the ternary diagram was analyzed using the centroid plot of three variables, of which the sum of the three variables was constant (ggplot2) (Bulgarelli et al., 2015).

## Results

### Metadata General Description

Using the Illumina HiSeq 2500 platform of 16S rRNA gene V4 region amplicons, a total of 460,679 sequences were obtained in seven samples of the red panda (**Supplementary Table S1**) with a median length of 253 bp. The number of sequences per sample ranged from 57,849 to 73,638. A total of 9,379 unique OTUs were obtained at 97% identity and an average of 1,340 OTUs for each sample, which range from 883 to 1643 (**Supplementary Figure S1A** and **Supplementary Table S1**). The number of OTUs was higher in the stomach (1643) and duodenum (1638). An average of 44 unclassified tags were observed in samples from the stomach (104 unclassified) and duodenum (121 unclassified) but not in the faecal samples. After the annotation of species through the SSU rRNA database, the taxonomic levels included kingdom, phylum, class, order, family, genus, and species of microbiota were conducted, which revealed in-depth microbial information (**Supplementary Figure S1B**). The large numbers of sequences in samples from the stomach and small intestine were detected at the species level. Samples from the large intestine revealed more sequences at the genus level. Species profiles observed from colon, rectum, and feces (**Supplementary Figure S2A**) tended to approach the saturated platform but also increased with samples from the stomach and small intestine. Arranging the rank abundance curve gave the same result and shows that only a large curve span is obtained from the colon, rectum, and feces (**Supplementary Figure S2B**). Generally, a large number of microbial sequences were investigated in the red panda, of which the number of the sequence is distinct among the GIT segments.

### Microbial Diversity Across the Red Panda GIT

**FIGURE 1:**
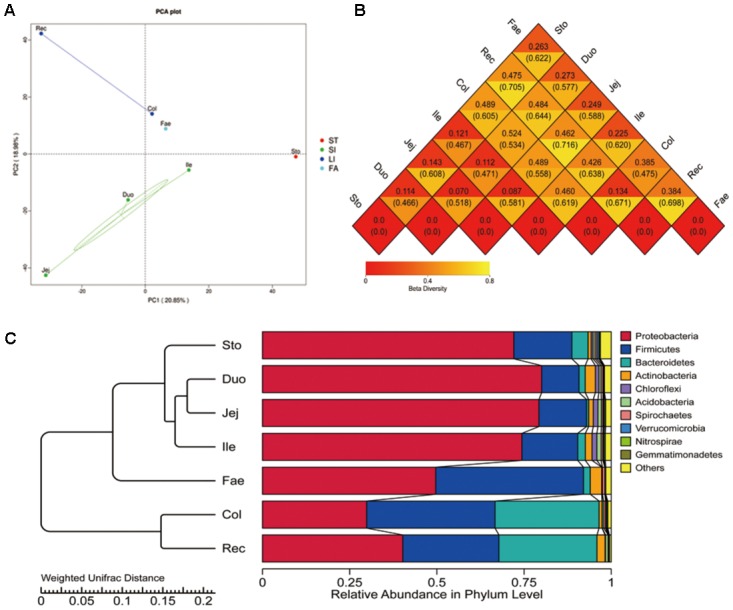
Beta diversity of microbiota in red panda GIT. **(A)** Principal component analysis (PCA) with unweighted uniFrac of bacterial community in GIT samples. **(B)** Heatmap of the distance matrix, number above and under the central line in each rhombus is calculated using the weighted uniFrac and unweighted uniFrac methods, with higher values showing significant differences. **(C)** Clustering of the microbiota at the phylum level. Sto, Duo, Jej, Ile, Col, Rec, and Fae represent samples from the stomach, duodenum, jejunum, ileum, colon, rectum, and faecal, respectively.

The alpha diversity (Observed species, Shannon, Simpson, Chao1, Ace, and goods coverage) was assessed by OTUs (**Supplementary Table S2**). Higher bacterial diversity was observed in both the large intestine and fecal samples than that in the stomach and small intestine. According to the ANOSIM analysis, significant differences were found in the bacterial community structure in the stomach, small intestine, large intestine, and faecal (*P* < 0.05). The number of species in the stomach and small intestine samples was higher than that of the large intestine and fecal samples. The beta diversity of the microbiota indicated that GIT segments are divided into four distinct groups: the stomach, small intestine, large intestine, and feces (**Figure 1A**). The heat map (weighted and unweighted uniFrac) of the distance matrix from the rectum and faecal samples showed a higher number than other samples (**Figure 1B**). This shows significant differences in the large intestine compared with other segments. A histogram generated by clustering analysis at phylum level divided the GIT samples into four major groups: the stomach, small intestine, large intestine, and feces (**Figure 1C**). Of the first three major bacterial phyla (Proteobacteria, Firmicutes, and Bacteroidetes), Proteobacteria predominated in all segments, especially in the stomach, small intestine and feces. Firmicutes was mainly distribute in faecal compared with other segments. Bacteroidetes was abundant in the large intestine.

### Distinct Microbiota Across the Red Panda GIT

**FIGURE 2:**
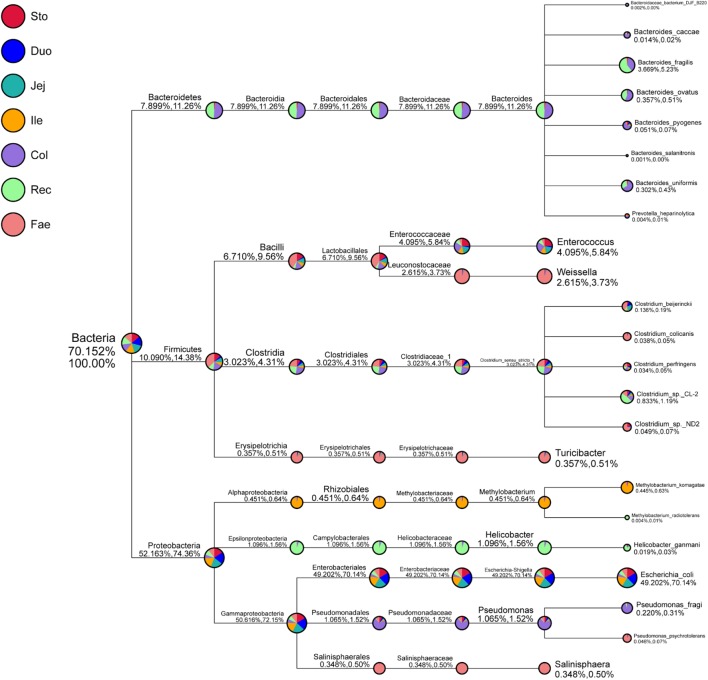
Specific species taxonomy tree analysis of microbiota in red panda GIT. Classification levels include the kingdom, phylum, class, order, family, genus, and species. The first percentile in parentheses shows the percentage of microflora in all the detected bacteria. The second percentile in parentheses shows the percentage of microflora in all the selected bacteria. Sto, Duo, Jej, Ile, Col, Rec, and Fae represent samples from the stomach, duodenum, jejunum, ileum, colon, rectum, and faecal, respectively.

Next, the classification of specific taxonomy groups of species (e.g., kingdom, phylum, class, order, family, genus, and species) was conducted (**Figure 2** and **Supplementary Figure S3**). Proteobacteria (52.16%), Firmicutes (10.09%), and Bacteroidetes (7.90%) were the three major GIT phyla. *Escherichia–Shigella* (49.20%), *Helicobacter* (1.10%), *Pseudomonas* (1.07%), *Methylobacterium* (0.45%), and *Salinisphaera* (0.35%) mainly comprised the phylum Proteobacteria. Moreover, *Escherichia–Shigella* mainly included higher abundances of *Escherichia coli* (49.20%). *Escherichia–Shigella* showed the closest relationship with *Proteus* and *Morganella* in the evolutionary tree (**Supplementary Figure S4**). Firmicutes were primarily composed of *Enterococcus* (4.10%), *Clostridium_sensu_ stricto_1* (3.02%), *Weissella* (2.62%), and *Turicibacter* (0.36%). The genus *Clostridium_sensu_stricto_1*, which mainly consisted *Clostridium_sp._CL-2* (0.83%), *Clostridium_beijerinckii* (0.14%), *Clostridium_*sp.*_*ND2 (0.05%), *Clostridium_colicanis* (0.04%), and *Clostridium_perfringens* (0.03%), was the closest to the genus *sarcina* in the evolutionary tree (**Supplementary Figure S4**). Bacteroidetes was mainly composed of *Bacteroides* (7.89%), including *Bacteroides_fragilis* (3.67%), *Bacteroides_ovatus* (0.36%), *Bacteroides_uniformis* (0.30%), *Bacteroides_pyogenes* (0.05%), and *Bacteroides_caccae* (0.01%). Moreover, *Bacteroides* was the closest genus to *Alloprevotella*, *Prevotella_7*, and *Prevotella_9* in the phylogenetic tree (**Supplementary Figure S4**). The species annotation of red panda GIT microbiota was further analyzed at the family, genus, and species level using the SSU rRNA database (**Supplementary Figures S1B**, **S3**, **S5**). The composition of the entire red panda GIT, Enterobacteriaceae, Enterococcaceae, *Escherichia–Shigella*, *Enterococcus*, and *Escherichia coli* were enriched in the stomach and small intestine. Bacteroidaceae, Peptostreptococcaceae, Helicobacteraceae, Lachnospiraceae, Ruminococcaceae, Pseudomonadaceae, *Bacteroides*, *Helicobacter*, and *Pseudomonas* were mainly in the large intestines. Leuconostocaceae, *Weissella*, *Salinisphaera*, and *Turicibacter* were major in the faecal.

**FIGURE 3:**
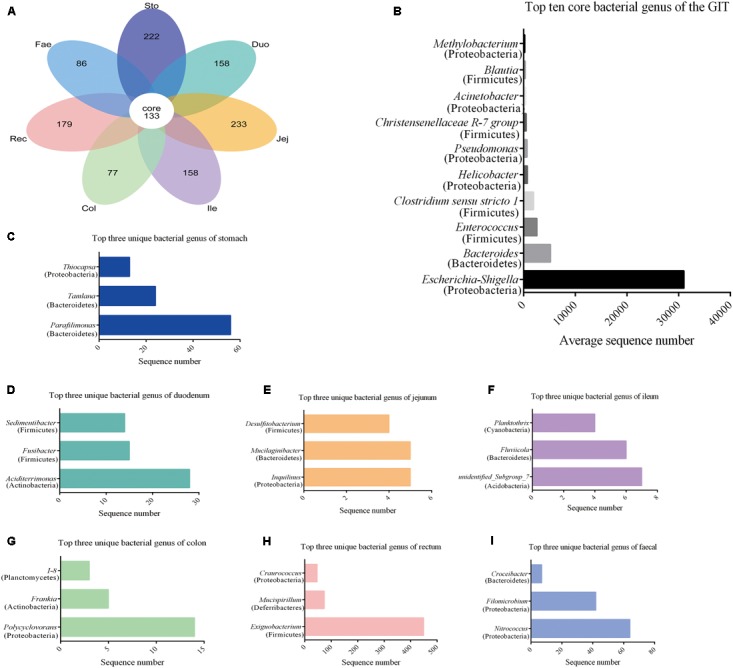
Core and unique microbiota in red panda GIT. **(A)** The venn petal diagrams of the bacterial community in GIT. **(B)** Top 10 core bacterial genera of all the segments. **(C–I)** Top three unique bacterial genera in samples from the stomach, duodenum, jejunum, ileum, colon, rectum, and faecal, respectively.

Using the Venn petal diagrams, a total of 133 core OTUs were obtained from the GIT samples of the red panda (**Figure 3A**). Of the 133 core OTUs, 100 genera of bacteria were identified. Of all the GIT samples, *Escherichia–Shigella* was the highest among the top 10 bacterial species, followed by *Bacteroides*, *Enterococcus*, *Clostridium_sensu_stricto_1*, *Helicobacter*, *Pseudomonas*, *Christensenellaceae* R-7 group, *Acinetobacter*, *Blautia*, and *Methylobacterium* (**Figure 3B** and **Supplementary Table S3**). The unique OTUs for stomach, duodenum, jejunum, ileum, colon, rectum, and faecal were 222, 158, 233, 158, 77, 179, and 86, respectively (**Figure 3A** and **Supplementary Table S4**). In the stomach sample, *Parafilimonas*, *Tamlana*, and *Thiocapsa* were the top three unique bacterial genera (**Figure 3C**). In the small intestine, *Aciditerrimonas*, *Inquilinus*, and unidentified*_ Subgroup_*7 were predominate in the duodenum, jejunum, and ileum, respectively (**Figures 3D–F**). *Polycyclovorans* and *Exiguobacterium* contribute most of the unique bacterial genus in colon and rectum samples. Moreover, *Nitrococcus*, *Filomicrobium*, and *Croceibacter* constituted the top three unique bacterial genera in faecal samples.

### Predominant Bacteria With Classification From Phylum to Genus Revealed in the GIT of the Red Panda

**FIGURE 4:**
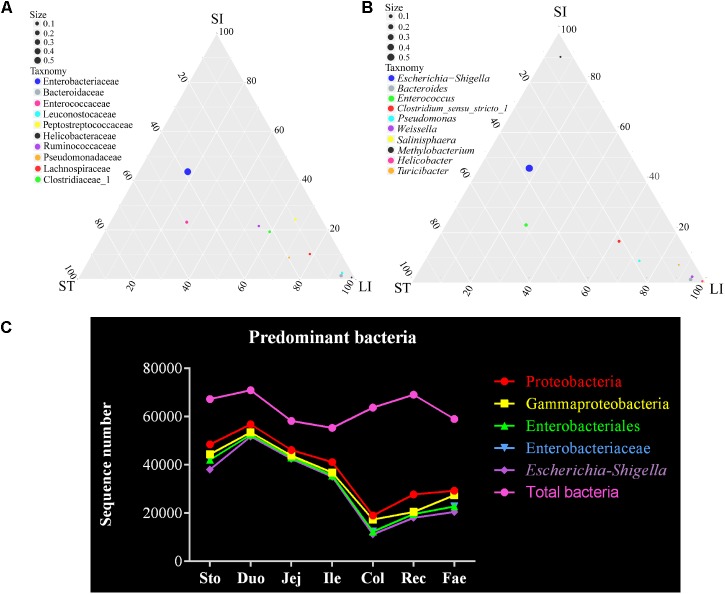
*Escherichia–Shigella* is a major genus of bacteria in the red panda GIT. **(A)** The ternary plot of bacterial between the stomach, small intestine, and large intestine at family level. The three vertices in the figure represent the sample group (ST: stomach; SI: small intestinal; LI: large intestinal). Circles represent species, and size represents relative abundance. The closer the circle is to the vertex, the higher the relative abundance of this species. **(B)**The ternary plot of bacterial between the stomach, small intestine, and large intestine at genus level. **(C)** Total bacteria and the major bacterial genera of *Escherichia–Shigella*, classified from the phylum to genus level across the GIT.

Finally, the dominant bacteria belonged to the phylum Proteobacteria were analyzed in the red panda’s GIT (**Figures 1**, **2**). The ternary plots of the bacteria of the stomach, small intestine, and large intestine samples at family and genus levels showed that the *Escherichia–Shigella* and Enterobacteriaceae were predominant and similar to the samples from the stomach and small intestine (**Figures 4A,B**). Bacteria sequenced due to the use of the 16S rRNA gene V4 region amplicons were less accurate. Thus, the statistical correlation of the *Escherichia–Shigella*, Enterobacteriaceae, Enterobacteriales, *Gammaproteobacteria*, and Proteobacteria across the GIT were exhibited in **Figure 4C**. These bacteria were present in the stomach and small intestine at a higher level than those in the large intestine and feces; the highest number was found in the duodenum. The duodenum bacterial sequence numbers for *Escherichia–Shigella*, Enterobacteriaceae, Enterobacteriales, *Gammaproteobacteria*, and Proteobacteria were 51,754, 52,527, 52,527, 53,525, and 56,810, respectively (**Supplementary Table S5**). We suggest that the bacteria are related to the small intestine, especially the duodenum.

## Discussion

Consistent with our hypothesis, the high-throughput sequencing data from the current study showed that the microbiota in the red panda GIT is distinct, with the Proteobacteria predominating (**Figure 3**). In healthy mammals, the stomach is the first segment of the GIT that receives and digests food and resides in bacteria that originated from Firmicutes, Bacteroidetes, and Proteobacteria (Gu et al., 2013; Gulino et al., 2013; Weldon et al., 2015). Moreover, the responsibility of nutrition digestion in small intestine is to ferment monosaccharides and amino acids (Gu et al., 2013). This gut nutrition environment is suitable for facultative anaerobes growth, which mainly belongs to Proteobacteria. This existence and “disappearance” of the hypothetical “transient microbiota” may explain the great number of bacteria in the stomach and small intestine of mice, especially the highest numbers of duodenum bacteria (Gu et al., 2013). Our results revealed that the microbiota in the stomach and the small intestine of red panda showed a large number of OTUs: 1643 OTUs in the stomach and 1638 OTUs in the small intestine, mostly Proteobacteria (**Supplementary Figure S1A** and **Supplementary Table S1**). The large intestine digests polysaccharides and shows the dominance of Bacteroidetes (Faith et al., 2013; Seedorf et al., 2014). Bacteroidetes dominated the samples from the large intestine of the red panda, which is consistent with our findings. Although the number of bacterial sequences in these studies is not exactly the same, they have similar trends. Our data indicates that Firmicutes is the dominant species with a percentage of 40.49% in the fecal sample, compared with other segments. These results are consistent with the findings from other studies on microbiota in wild and captive red panda faecal (Kong et al., 2014; Li et al., 2015; Williams et al., 2018). In Williams’s study, they used the Illumina MiSeq method to test the bacteria 16S rRNA V3–V4 region of two captive red panda faecal at different stages of weaning. Their results show that Firmicutes are the most abundant bacteria, with a percentage of 71 ± 6.9%. The Illumina MiSeq and Illumina HiSeq are two platforms widely used in the bacteria DNA sequencing, and they have 150 and 100 bp paired-end reads, respectively (Caporaso et al., 2012). The selection of the bacterial target regions of 16S rRNA can lead to the error and bias in amplicon-based microbial community (Gohl et al., 2016; Sinha et al., 2017). Nonetheless, these approaches allow the use of MiSeq and HiSeq methods to study the same trends of microbial flora.

For wild animals, bacterial communities in the feces are the easiest to study using living wild animals (Kong et al., 2014; Barelli et al., 2015; Li et al., 2015; Borbón-García et al., 2017; McKenney et al., 2017; Menke et al., 2017). The study of GIT microbiota has broadened our understanding of host digestion, immune response, physiology and disease treatment and will help us further develope better ways of protecting wild animals (Amato, 2013; Bahrndorff et al., 2016; Stumpf et al., 2016). In recent years, some studies have focused on the study of the gastrointestinal flora of wild mammals. For example, when analyzing the V4 region of the 16S rRNA gene sequenced by Illumina MiSeq, the data showed that the colonized bacteria of macaques GIT are mainly Firmicutes, Bacteroidetes, Spirochaetes, and Proteobacteria (Yasuda et al., 2015). However, our study found that Proteobacteria is the major bacteria in the red panda GIT, followed by Firmicutes and Bacteroidetes (**Figure 1C**). Moreover, different results were found in the red kangaroo (*Macropus rufus*) GIT; Firmicutes, Bacteroidetes, and Actinobacteria were the major bacteria, and the target regions for 16S rRNA genes V3 and V4 were analyzed by Illumina MiSeq (Li et al., 2016). In addition to different sequencing methods, different breeds of animals contribute most of these differences. Similarly, Bacteroidetes (24.64%) and Firmicutes (13.28%) are mainly characterized in the GIT of a Brazilian Nelore steer. A similar result was found in the GIT of the bison (Bergmann, 2017), with the Bacteroidetes abundant in most segments. However, in the GIT of dairy cattle, the first three major bacteria were Firmicutes (42.22%), Bacteroidetes (21.00%), and Proteobacteria (17.56%). Bacterial relative abundance was found contributed most of the differences among different species of animals than that of the taxa of the bacteria (Chen et al., 2017). Firmicutes were found to be the dominant bacteria in the feces of primates (gibbon, golden monkey, chimpanzee, and assam macaque) and Proteobacteria were the dominant bacteria in carnivora (red panda, giant panda, tiger, black bear, and lion). Consistent with this study, our study found that Proteobacteria is primarily responsible for the red panda GIT, especially in the stomach and small intestine.

Our previous study showed that the predominate DGGE band in the GIT of the red panda was identified closest to the *Escherichia coli* strain KR-1. The same trend was found in this study. Proteobacteria was the main phyla in the GIT of red panda, including the class Gammaproteobacteria, the order Enterobacteriales, the family Enterobacteriaceae, and the genus *Escherichia–Shigella* (**Figure 4**). Microbiota appear to be functionally stable in the gut of different healthy hosts (Costea et al., 2017; Mehta et al., 2018). A recent study found that Proteobacteria (59.00%) is a dominant factor that influences functional variability in human gut microbiota than that in Bacteroidetes (12.00%) and Firmicutes (29.00%) (Bradley and Pollard, 2017). The class Gammaproteobacteria and Betaproteobacteria were found to be two of the four core microbiome in a survey of 112 animal species (herbivores, omnivores, and carnivores) representing 14 mammalian orders (Nishida and Ochman, 2017). However, Gammaproteobacteria was observed more abundantly in captive animals in a study of the gut microbiome in 41 mammalian taxa (herbivores, omnivores, and carnivores) across six orders (Mckenzie et al., 2017). Despite this, Betaproteobacteria was found to be more abundant in wild animals in the six mammalian orders. This shift in Proteobacteria bacteria can be inferred to be related to animal diets, species and whether they are captive or wild. Moreover, Proteobacteria abundance seems to be sensitive to environmental changes. For example, Proteobacteria dominates in studies of fecal matter in brown bears (*Ursus arctos*) (Sommer et al., 2016) and Andean bears (Borbón-García et al., 2017). The change from winter to summer, frequent activities and food intake can lead to an increasing abundance of Proteobacteria in the feces of brown bears (*Ursus arctos*). Although captivity plays an important role in the population breeding and species conservation of wildlife, the bacterial diversity in their feces has declined (e.g., Andean bear, red panda, Przewalski’s horse, woodrats, and panda) (Kohl et al., 2014; Kong et al., 2014; Wei et al., 2015; Borbón-García et al., 2017; Metcalf et al., 2017). These studies show that the protection of indigenous microbiota in the gut of wild animals is another important aspect of human conservation of wildlife. Currently, within its usefulness, *Escherichia–Shigella* can digest and absorb animal food. For example, *Escherichia–Shigella* is the dominant genus of feces in two captive red pandas during weaning (Williams et al., 2018). Moreover, *Escherichia–Shigella*, *Clostridium*, *Turicibacter*, and *Streptococcus* are the major genera in the wild giant panda feces at different times of the year, especially the utilization of the shoot and leaf stage (Wu et al., 2017). Consistent with this trend, *Escherichia–Shigella* is abundant in leaves that are predominantly mucus-seasoned samples (Williams et al., 2016). However, the overgrowth of Proteobacteria can also lead to some diseases, such as inflammatory bowel disease (Mukhopadhya et al., 2012) and metabolic syndrome (Shin et al., 2015). Additionally, *Escherichia–Shigella* is found to be closely related to *Proteus* and *Morganella* in the evolutionary tree in our study (**Figure 4C** and **Supplementary Figure S4**). A previous study shows that *Proteus* is associated with Crohn’s disease (Lopetuso et al., 2018). With the distemper virus infection, the number of dominant *Escherichia* is reduced (Zhao et al., 2017). Based on the relatively few research results, we cannot confirm the role of the *Escherichia–Shigella* in red panda. However, recent studies have shown that different gastrointestinal (GI) diseases result in the significantly different composition of gut microbiota (Lopetuso et al., 2018). As for adult and old red pandas, gastrointestinal (e.g., ulceration, esophagitis, gastritis, diaphragmatic hernia, intussusception, and gastric torsion) and renal diseases (e.g., chronic interstitial nephritis and renal cysts) are mainly responsible for animal deaths (Delaski et al., 2015). Moreover, captive red pandas suffer from clinical illnesses, such as infectious diseases and parasites (Philippa and Ramsay, 2011). Gut microbes, such as *Clostridium*, *Lactobacillus*, *Eggerthella*, and *Bacteroides*, are usually active during the decomposing corpses (6–9 days) of dead bodies under natural conditions (Hyde et al., 2013). Although the GIT samples in our study are not fresh, they were collected within one day of the animal’s death. Therefore, we consider that death status did not have significant impact on intestinal flora in the dead red panda in our study. Despite this, few intestinal samples of the dead red panda are available for further study, such as microbial culture and metabolomics studies. Not enough intestinal microflora information is available in other red panda studies for comparison. Given the similar habitat, dietary, and species evolution, the comparison of gut microbiota in other wildlife, such as the giant panda, is crucial to the GIT microbiota of red panda.

## Conclusion

The contributions of this work are presented as follows: our study provides a first preliminary understanding of the biogeography of GIT microbiota in the red panda (*Ailurus fulgens*). Four different bacterial community areas, namely, the stomach, small intestine, large intestine, and feces, were obtained. Proteobacteria, Firmicutes, and Bacteroidetes were dominated by red panda’s GIT. It will be important that future research investigate the microbial culture, metagenomics and metabolism of red panda GIT, especially in *Escherichia–Shigella*. Additionally, the results of the microbiota of the red panda in our study are limited. In the future, it will be necessary to conduct in-depth comparative analysis with other wild animals.

## Author Contributions

YaZ, XN, DZ, JD, LN, and YP conceived and designed the project. YiZ, YL, YcL, SX, and MZ for sample collection. YaZ, YL, QL, and LX performed experiments. YiZ, YL, KP, and BJ analysis and interpretation the data. YaZ, DZ, and XN wrote the manuscript. YaZ, YiZ, and DZ contributed to the manuscript equally. YiZ and XN revised the manuscript. All authors read and approved the final manuscript.

## Conflict of Interest Statement

The authors declare that the research was conducted in the absence of any commercial or financial relationships that could be construed as a potential conflict of interest.

## Footnotes

1 https://www.ncbi.nlm.nih.gov/bioproject/PRJNA385220

2 http://ccb.jhu.edu/software/FLASH/

3 http://qiime.org/scripts/split_libraries_fastq.html

4 http://drive5.com/uchime/uchime_download.html

5 http://www.drive5.com/usearch/manual/uchime_algo.html

6 http://drive5.com/uparse/

7 http://www.arb-silva.de/

8 http://www.drive5.com/muscle/

## Supplementary Material

FIGURE S1 Tag information and bacterial classification. **(A)** Total tags, taxon tags, operational taxonomic units (OTUs), unclassified tags, and unique tags. **(B)** The classification level includes the kingdom, phylum, class, order, family, genus, and species.

FIGURE S2 Rarefaction curve analysis. Duplicate samples of OTU subgroups are evaluated whether further sampling would likely yield additional taxa, as indicated by the plateau value. **(A)** The y-axis represents the number of OTUs detected and the x-axis indicates the number of taxa in the analyzed subsets of sequences. **(B)** Rank abundance curves were used to estimate the richness and evenness of taxa present in the samples. The y-axis indicates the relative abundance of OTUs and the x-axis indicates the number of OTUs according to the relative abundance from largest to smallest. The larger the span curve is on the x-axis, the higher the species richness. The smoother the curve on the y-axis, the more evenly the species are distributed.

FIGURE S3 Species-specific tree analysis of bacteria from the **(A)** stomach, **(B)** duodenum, **(C)** jejunum, **(D)** ileum, **(E)** colon, **(F)** rectum, and **(G)** faecal. The first percentile in brackets shows the percentage of all detected bacteria in the microbiota. The second percentile in parentheses shows the percentage of microbial communities in all selected bacteria.

FIGURE S4 Top 100 bacteria genus in the evolutionary tree of red panda GIT.

FIGURE S5 Species annotation of microbiota of red panda GIT at levels from **(A)** phylum, **(B)** class, **(C)** order, **(D)** family, and **(E)** genus.

TABLE S1 General information of sequence data. Sto, Duo, Jej, Ile, Col, Rec, and Fae represent samples from the stomach, duodenum, jejunum, ileum, colon, rectum, and faecal, respectively.

TABLE S2 Alpha diversity index, including observed species, Shannon, Simpson, chao1, ACE, and goods coverage. Sto, Duo, Jej, Ile, Col, Rec, and Fae represent samples from the stomach, duodenum, jejunum, ileum, colon, rectum, and faecal, respectively.

TABLE S3 Core bacterial average sequence in red panda GIT. Sto, Duo, Jej, Ile, Col, Rec, and Fae represent samples from the stomach, duodenum, jejunum, ileum, colon, rectum, and faecal, respectively.

TABLE S4 Unique bacterial genus from the stomach, duodenum, jejunum, ileum, colon, rectum, and faecal samples, respectively. Sto, Duo, Jej, Ile, Col, Rec, and Fae represent samples from the stomach, duodenum, jejunum, ileum, colon, rectum, and faecal, respectively.

TABLE S5 Dominant bacterial community at the classification level from phylum to genus in red panda GIT.
