# Supplementary figures and images for: Microbial Biogeography Along the Gastrointestinal Tract of a Red Panda
Source: Front Microbiol. 2018 Jul 5;9:1411. doi: 10.3389/fmicb.2018.01411 (PMC6042058; doi:10.3389/fmicb.2018.01411)

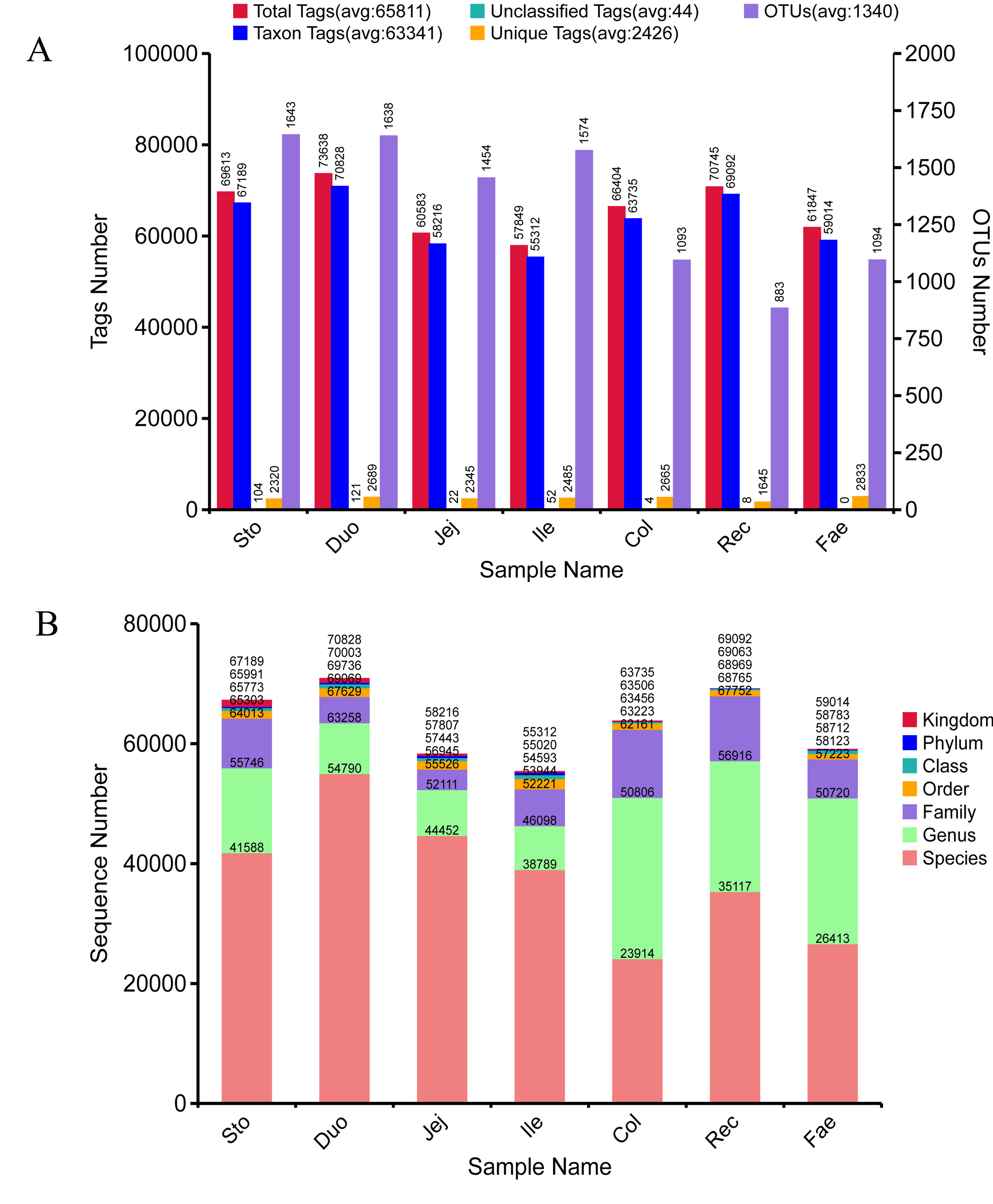

Supplement: FIGURE S1 — Tag information and bacterial classification. (A) Total tags, taxon tags, operational taxonomic units (OTUs), unclassified tags, and unique tags. (B) The classification level includes the kingdom, phylum, class, order, family, genus, and species. [file Image_1.TIF]

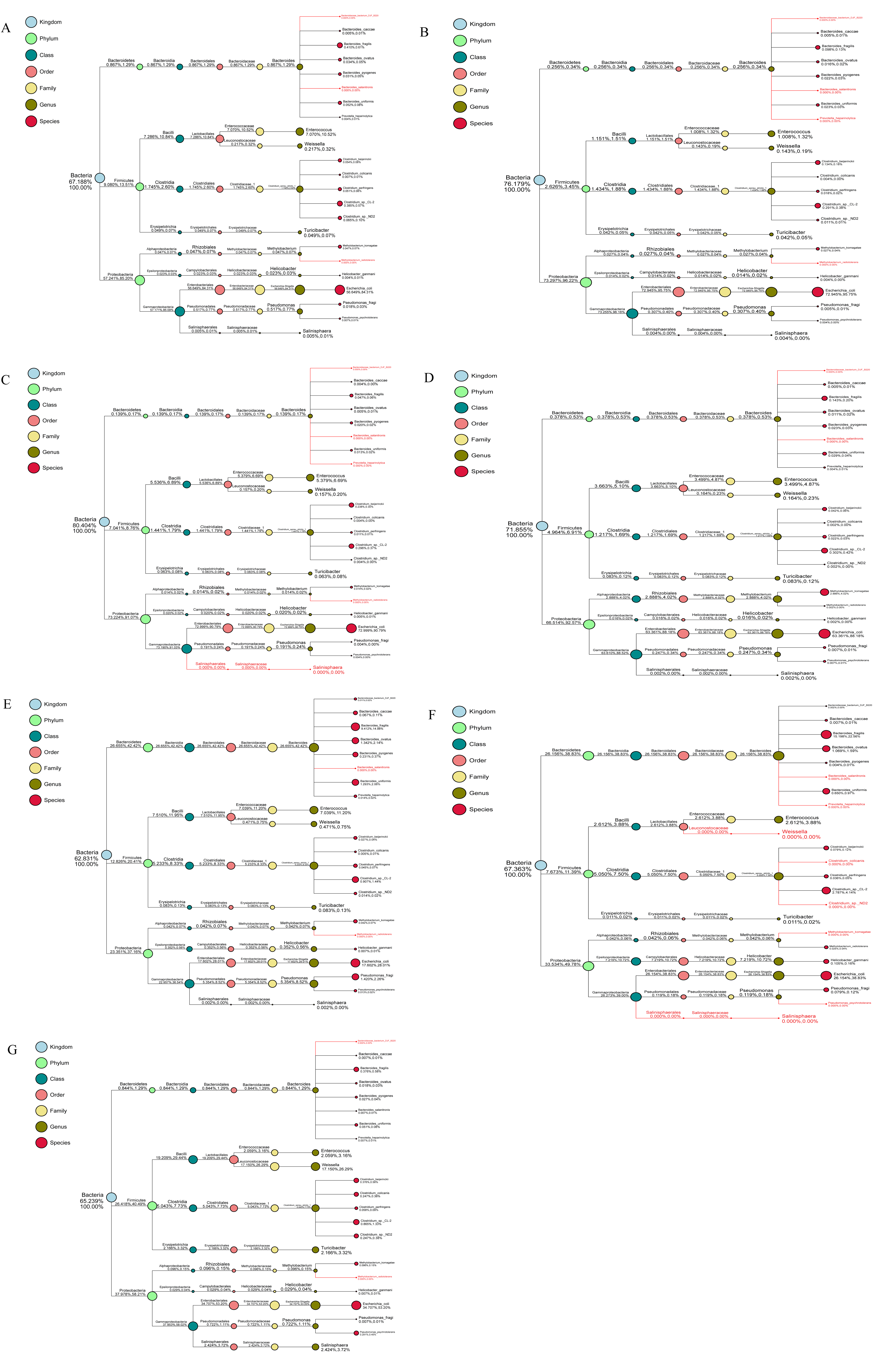

Supplement: FIGURE S3 — Species-specific tree analysis of bacteria from the (A) stomach, (B) duodenum, (C) jejunum, (D) ileum, (E) colon, (F) rectum, and (G) faecal. The first percentile in brackets shows the percentage of all detected bacteria in the microbiota. The second percentile in parentheses shows the percentage of microbial communities in all selected bacteria. [file Image_3.TIF]

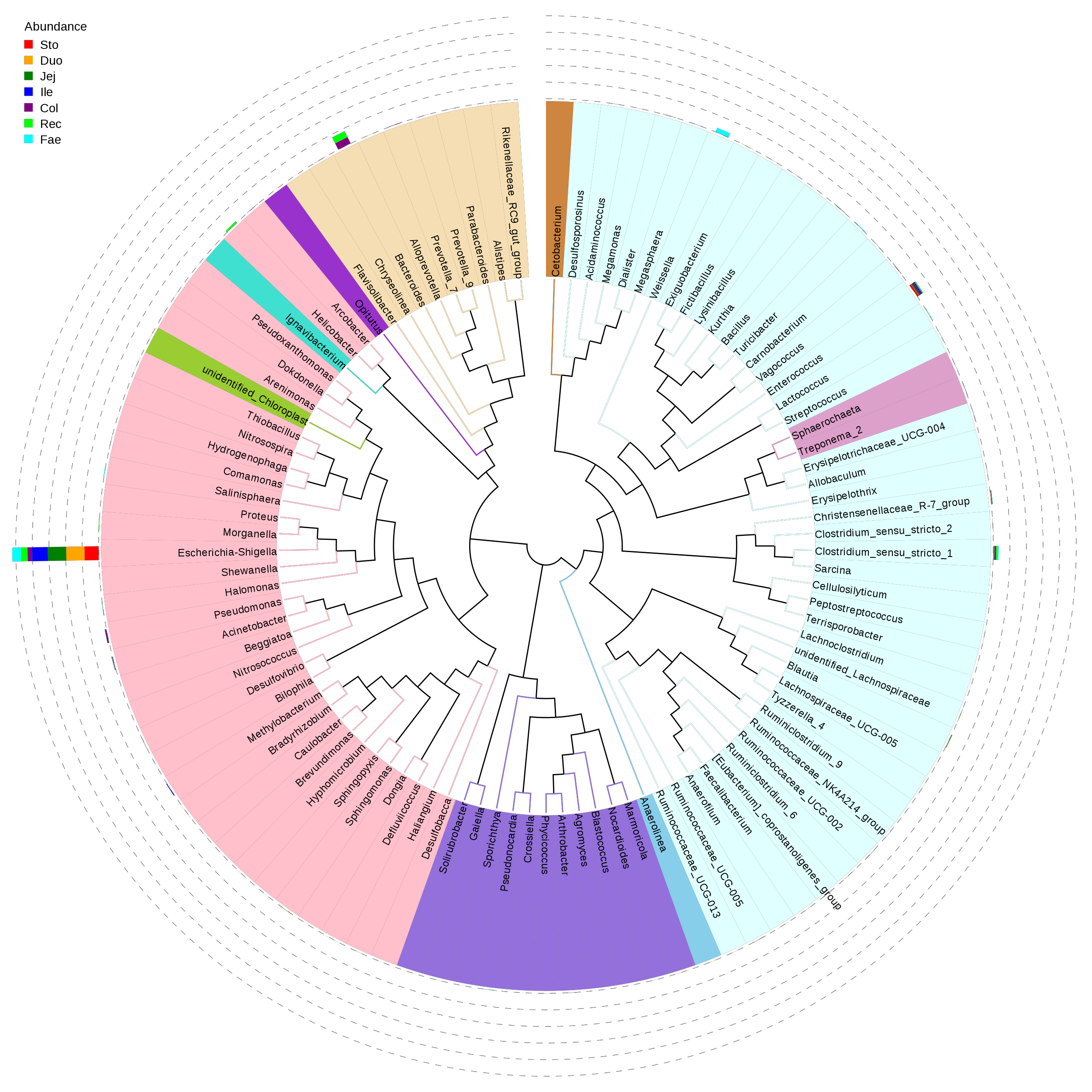

Supplement: FIGURE S4 — Top 100 bacteria genus in the evolutionary tree of red panda GIT. [file Image_4.TIF]

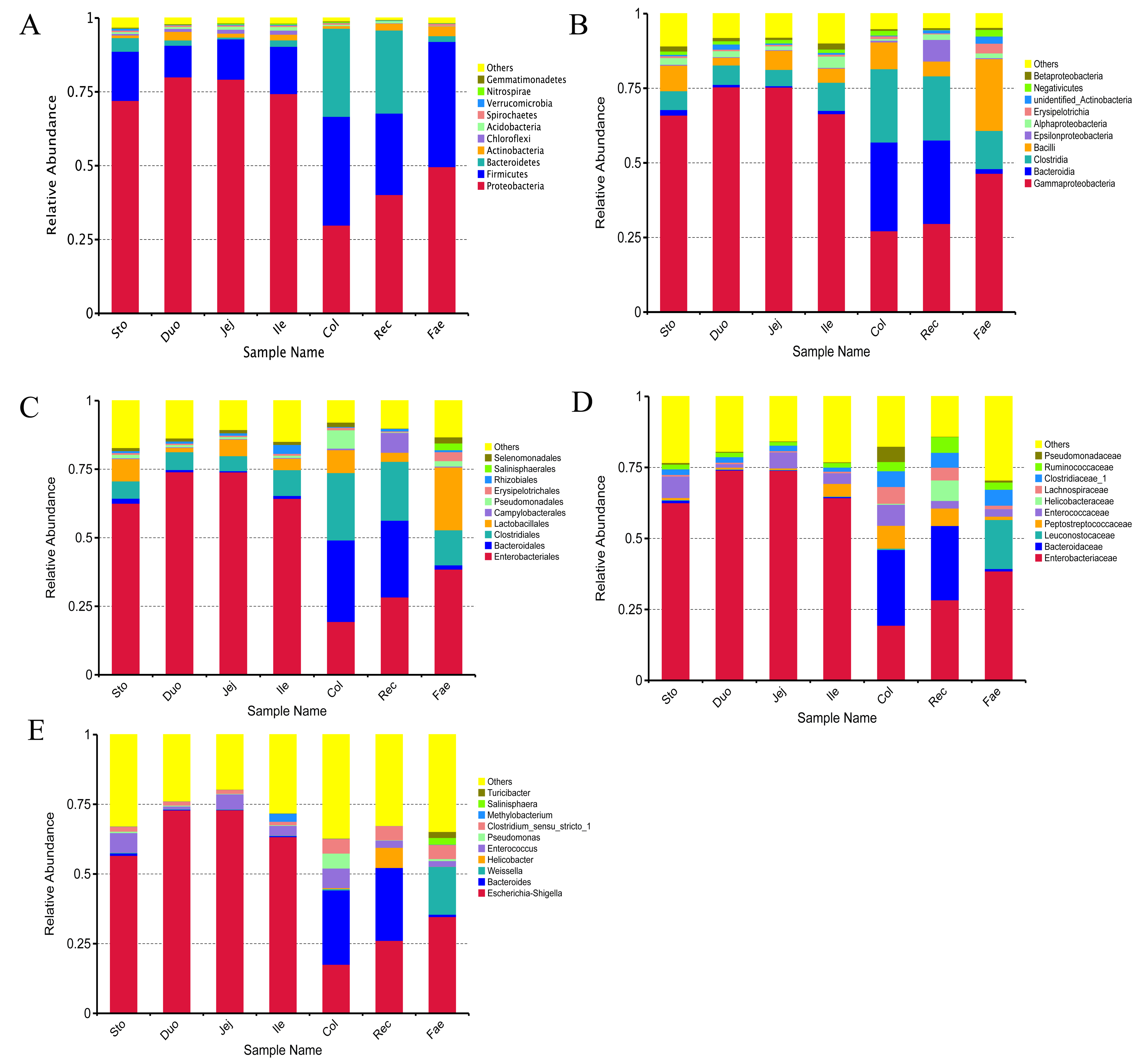

Supplement: FIGURE S5 — Species annotation of microbiota of red panda GIT at levels from (A) phylum, (B) class, (C) order, (D) family, and (E) genus. [file Image_5.TIF]
