# Supplementary material for: Microbial Biogeography Along the Gastrointestinal Tract of a Red Panda
Source: Front Microbiol. 2018 Jul 5;9:1411. doi: 10.3389/fmicb.2018.01411 (PMC6042058; doi:10.3389/fmicb.2018.01411)
Supplement: TABLE S1 — General information of sequence data. Sto, Duo, Jej, Ile, Col, Rec, and Fae represent samples from the stomach, duodenum, jejunum, ileum, colon, rectum, and faecal, respectively. [file Table_1.DOC]

**Table S1.** General information of sequence data. Sto, Duo, Jej, Ile, Col, Rec, and Fae represent samples from the stomach, duodenum, jejunum, ileum, colon, rectum, and faecal, respectively.

| **Sample Name** | **Barcode Sequence** | **Raw Tags** | **Clean Tags** | **Effective Tags** | **Average length (bp)** | **Effective**  **(%)** | **Unclassified Tag** | **Unique Tag** | **OTU number** |
| --- | --- | --- | --- | --- | --- | --- | --- | --- | --- |
| Sto | CATTTT,GATCAG | 73,385 | 72,638 | 69,613 | 253 | 94.86 | 104 | 2320 | 1643 |
| Duo | CATTTT,TAGCTT | 76,016 | 75,243 | 73,638 | 253 | 96.87 | 121 | 2689 | 1638 |
| Jej | CATTTT,GGCTAC | 63,133 | 62,544 | 60,583 | 253 | 95.96 | 22 | 2345 | 1454 |
| Ile | CATTTT,CTTGTA | 68,361 | 67,681 | 57,849 | 253 | 84.62 | 52 | 2485 | 1574 |
| Col | CATTTT,AGTCAA | 72,295 | 71,661 | 66,404 | 253 | 91.85 | 4 | 2665 | 1093 |
| Rec | CATTTT,AGTTCC | 73,307 | 72,627 | 70,745 | 253 | 96.51 | 8 | 1645 | 883 |
| Fae | CATTTT,ATGTCA | 71,383 | 70,799 | 61,847 | 253 | 86.64 | 0 | 2833 | 1094 |
