# Supplementary material for: Microbial Biogeography Along the Gastrointestinal Tract of a Red Panda
Source: Front Microbiol. 2018 Jul 5;9:1411. doi: 10.3389/fmicb.2018.01411 (PMC6042058; doi:10.3389/fmicb.2018.01411)
Supplement: TABLE S2 — Alpha diversity index, including observed species, Shannon, Simpson, chao1, ACE, and goods coverage. Sto, Duo, Jej, Ile, Col, Rec, and Fae represent samples from the stomach, duodenum, jejunum, ileum, colon, rectum, and faecal, respectively. [file Table_2.DOC]

**Table S2.** Alpha diversity index, including observed species, Shannon, Simpson, chao1, ACE, and goods coverage. Sto, Duo, Jej, Ile, Col, Rec, and Fae represent samples from the stomach, duodenum, jejunum, ileum, colon, rectum, and faecal, respectively.

| **Sample Name** | **Observed Species** | **Shannon** | **Simpson** | **Chao1** | **ACE** | **Goods Coverage** |
| --- | --- | --- | --- | --- | --- | --- |
| Sto | 1475 | 4.208 | 0.671 | 1609.561 | 1662.285 | 0.995 |
| Duo | 1445 | 3.202 | 0.467 | 1627.194 | 1662.894 | 0.994 |
| Jej | 1302 | 2.938 | 0.464 | 1437.72 | 1470.655 | 0.995 |
| Ile | 1574 | 3.769 | 0.594 | 2424.268 | 2103.943 | 0.99 |
| Col | 936 | 5.318 | 0.925 | 1046.519 | 1059.936 | 0.997 |
| Rec | 817 | 4.714 | 0.888 | 885.546 | 895.035 | 0.998 |
| Fae | 962 | 4.952 | 0.846 | 1085.435 | 1097.937 | 0.997 |
