# Supplementary material for: Microbial Biogeography Along the Gastrointestinal Tract of a Red Panda
Source: Front Microbiol. 2018 Jul 5;9:1411. doi: 10.3389/fmicb.2018.01411 (PMC6042058; doi:10.3389/fmicb.2018.01411)
Supplement: TABLE S3 — Core bacterial average sequence in red panda GIT. Sto, Duo, Jej, Ile, Col, Rec, and Fae represent samples from the stomach, duodenum, jejunum, ileum, colon, rectum, and faecal, respectively. [file Table_3.DOC]

**Table S3.** Core bacterial average sequence in red panda GIT. Sto, Duo, Jej, Ile, Col, Rec, and Fae represent samples from the stomach, duodenum, jejunum, ileum, colon, rectum, and faecal, respectively.

| **Top ten core Bacterial Name** | **Sto** | **Duo** | **Jej** | **Ile** | **Col** | **Rec** | **Fae** | **Mean Value** |
| --- | --- | --- | --- | --- | --- | --- | --- | --- |
| k__Bacteria;p__Proteobacteria;c__Gammaproteobacteria;o__Enterobacteriales;f__Enterobacteriaceae;g__***Escherichia-Shigella***; | 38121 | 51754 | 42513 | 35078 | 11219 | 18073 | 20482 | 31034 |
| k__Bacteria;p__Bacteroidetes;c__Bacteroidia;o__Bacteroidales;f__Bacteroidaceae;g__***Bacteroides***; | 579 | 185 | 81 | 211 | 16983 | 18075 | 496 | 5230 |
| k__Bacteria;p__Firmicutes;c__Bacilli;o__Lactobacillales;f__Enterococcaceae;g__***Enterococcus***; | 4757 | 716 | 3133 | 1937 | 4487 | 1805 | 1216 | 2579 |
| k__Bacteria;p__Firmicutes;c__Clostridia;o__Clostridiales;f__Clostridiaceae_1;g__***Clostridium_sensu_stricto_1***; | 1173 | 1022 | 837 | 672 | 3335 | 3490 | 2978 | 1930 |
| k__Bacteria;p__Proteobacteria;c__Epsilonproteobacteria;o__Campylobacterales;f__Helicobacteraceae;g__***Helicobacter***; | 16 | 10 | 11 | 10 | 224 | 4991 | 18 | 754 |
| k__Bacteria;p__Proteobacteria;c__Gammaproteobacteria;o__Pseudomonadales;f__Pseudomonadaceae;g__***Pseudomonas***; | 347 | 215 | 113 | 136 | 3412 | 84 | 428 | 676 |
| k__Bacteria;p__Firmicutes;c__Clostridia;o__Clostridiales;f__Christensenellaceae;g__***Christensenellaceae_R-7_group***; | 512 | 632 | 439 | 445 | 671 | 6 | 791 | 499 |
| k__Bacteria;p__Proteobacteria;c__Gammaproteobacteria;o__Pseudomonadales;f__Moraxellaceae;g__***Acinetobacter***; | 464 | 352 | 304 | 330 | 833 | 50 | 747 | 440 |
| k__Bacteria;p__Firmicutes;c__Clostridia;o__Clostridiales;f__Lachnospiraceae;g__***Blautia***; | 37 | 27 | 20 | 29 | 863 | 1346 | 56 | 340 |
| k__Bacteria;p__Proteobacteria;c__Alphaproteobacteria;o__Rhizobiales;f__Methylobacteriaceae;g__***Methylobacterium***; | 32 | 21 | 9 | 1599 | 27 | 27 | 57 | 253 |
