# Supplementary material for: Microbial Biogeography Along the Gastrointestinal Tract of a Red Panda
Source: Front Microbiol. 2018 Jul 5;9:1411. doi: 10.3389/fmicb.2018.01411 (PMC6042058; doi:10.3389/fmicb.2018.01411)
Supplement: TABLE S4 — Unique bacterial genus from the stomach, duodenum, jejunum, ileum, colon, rectum, and faecal samples, respectively. Sto, Duo, Jej, Ile, Col, Rec, and Fae represent samples from the stomach, duodenum, jejunum, ileum, colon, rectum, and faecal, respectively. [file Table_4.DOC]

**Table S4.** Unique bacterial genus from the stomach, duodenum, jejunum, ileum, colon, rectum, and faecal samples, respectively. Sto, Duo, Jej, Ile, Col, Rec, and Fae represent samples from the stomach, duodenum, jejunum, ileum, colon, rectum, and faecal, respectively.

| **GIT** | **Sequencs number** | **Bacterial Name** |
| --- | --- | --- |
| Sto | 56 | k__Bacteria;p__Bacteroidetes;c__Sphingobacteriia;o__Sphingobacteriales;f__Chitinophagaceae;g__***Parafilimonas***; |
|  | 24 | k__Bacteria;p__Bacteroidetes;c__Flavobacteriia;o__Flavobacteriales;f__Flavobacteriaceae;g__***Tamlan****a*; |
|  | 13 | k__Bacteria;p__Proteobacteria;c__Gammaproteobacteria;o__Chromatiales;f__Chromatiaceae;g__***Thiocapsa***; |
| Duo | 28 | k__Bacteria;p__Actinobacteria;c__Acidimicrobiia;o__Acidimicrobiales;f__unidentified_Acidimicrobiales;g__***Aciditerrimonas***; |
|  | 15 | k__Bacteria;p__Firmicutes;c__Clostridia;o__Clostridiales;f__Family_XII;g__***Fusibacter***; |
|  | 14 | k__Bacteria;p__Firmicutes;c__Clostridia;o__Clostridiales;f__Family_XI;g__***Sedimentibacter***; |
| Jej | 5 | k__Bacteria;p__Proteobacteria;c__Alphaproteobacteria;o__Rhodospirillales;f__Rhodospirillaceae;g__***Inquilinus***; |
|  | 5 | k__Bacteria;p__Bacteroidetes;c__Sphingobacteriia;o__Sphingobacteriales;f__Sphingobacteriaceae;g__***Mucilaginibacter***; |
|  | 4 | k__Bacteria;p__Firmicutes;c__Clostridia;o__Clostridiales;f__Peptococcaceae;g__***Desulfitobacterium***; |
| Ile | 7 | k__Bacteria;p__Acidobacteria;c__Holophagae;o__Subgroup_7;f__unidentified_Subgroup_7;g__***unidentified_Subgroup_7***; |
|  | 6 | k__Bacteria;p__Bacteroidetes;c__Flavobacteriia;o__Flavobacteriales;f__Cryomorphaceae;g__***Fluviicola***; |
|  | 4 | k__Bacteria;p__Cyanobacteria;c__unidentified_Cyanobacteria;o__SubsectionIII;f__FamilyI;g__***Planktothrix***; |
| Col | 14 | k__Bacteria;p__Proteobacteria;c__Gammaproteobacteria;o__Xanthomonadales;f__Solimonadaceae;g__***Polycyclovorans***; |
|  | 5 | k__Bacteria;p__Actinobacteria;c__unidentified_Actinobacteria;o__Frankiales;f__Frankiaceae;g__***Frankia***; |
|  | 3 | k__Bacteria;p__Planctomycetes;c__Phycisphaerae;o__Phycisphaerales;f__Phycisphaeraceae;g__***I-8***; |
| Rec | 449 | k__Bacteria;p__Firmicutes;c__Bacilli;o__Bacillales;f__Family_XII;g__***Exiguobacterium***; |
|  | 74 | k__Bacteria;p__Deferribacteres;c__unidentified_Deferribacteres;o__Deferribacterales;f__Deferribacteraceae;g__***Mucispirillum***; |
|  | 47 | k__Bacteria;p__Proteobacteria;c__Alphaproteobacteria;o__Rhodospirillales;f__Acetobacteraceae;g__***Craurococcus***; |
| Fae | 66 | k__Bacteria;p__Proteobacteria;c__Gammaproteobacteria;o__Chromatiales;f__Ectothiorhodospiraceae;g__***Nitrococcus***; |
|  | 42 | k__Bacteria;p__Proteobacteria;c__Alphaproteobacteria;o__Rhizobiales;f__Hyphomicrobiaceae;g__***Filomicrobium***; |
|  | 7 | k__Bacteria;p__Bacteroidetes;c__Flavobacteriia;o__Flavobacteriales;f__Flavobacteriaceae;g__***Croceibacter***; |
