# Supplementary material for: Microbial Biogeography Along the Gastrointestinal Tract of a Red Panda
Source: Front Microbiol. 2018 Jul 5;9:1411. doi: 10.3389/fmicb.2018.01411 (PMC6042058; doi:10.3389/fmicb.2018.01411)
Supplement: TABLE S5 — Dominant bacterial community at the classification level from phylum to genus in red panda GIT. [file Table_5.doc]

**Table S5.** Dominant bacterial community at the classification level from phylum to genus in red panda GIT.

| **GIT** | **Proteobacteria** | **Gammaproteobacteria** | **Enterobacteriales** | **Enterobacteriaceae** | **Escherichia-Shigella** |
| --- | --- | --- | --- | --- | --- |
| Sto | 48494 | 44411 | 42106 | 42106 | 38121 |
| Duo | 56810 | 53525 | 52527 | 52527 | 51754 |
| Jej | 46178 | 43914 | 43088 | 43088 | 42513 |
| Ile | 41190 | 36815 | 35623 | 35623 | 35078 |
| Col | 19048 | 17365 | 12393 | 12393 | 11219 |
| Rec | 27798 | 20527 | 19615 | 19615 | 18073 |
| Fae | 29291 | 27448 | 22747 | 22747 | 20482 |
